# Apocynin and Diphenyleneiodonium Induce Oxidative Stress and Modulate PI3K/Akt and MAPK/Erk Activity in Mouse Embryonic Stem Cells

**DOI:** 10.1155/2016/7409196

**Published:** 2015-12-14

**Authors:** Jan Kučera, Lucia Binó, Kateřina Štefková, Josef Jaroš, Ondřej Vašíček, Josef Večeřa, Lukáš Kubala, Jiří Pacherník

**Affiliations:** ^1^Institute of Experimental Biology, Faculty of Science, Masaryk University, Kotlářská 267/2, 61137 Brno, Czech Republic; ^2^Institute of Biophysics, Academy of Sciences of the Czech Republic, Královopolská 2590/135, 61200 Brno, Czech Republic; ^3^Department of Histology and Embryology, Faculty of Medicine, Masaryk University, Kamenice 753/5, 62500 Brno, Czech Republic; ^4^International Clinical Research Center, Center of Biomolecular and Cellular Engineering, St. Anne's University Hospital, Pekařská 53, 65691 Brno, Czech Republic

## Abstract

Reactive oxygen species (ROS) are important regulators of cellular functions. In embryonic stem cells, ROS are suggested to influence differentiation status. Regulated ROS formation is catalyzed primarily by NADPH-dependent oxidases (NOXs). Apocynin and diphenyleneiodonium are frequently used inhibitors of NOXs; however, both exhibit uncharacterized effects not related to NOXs inhibition. Interestingly, in our model of mouse embryonic stem cells we demonstrate low expression of NOXs. Therefore we aimed to clarify potential side effects of these drugs. Both apocynin and diphenyleneiodonium impaired proliferation of cells. Surprisingly, we observed prooxidant activity of these drugs determined by hydroethidine. Further, we revealed that apocynin inhibits PI3K/Akt pathway with its downstream transcriptional factor Nanog. Opposite to this, apocynin augmented activity of canonical Wnt signaling. On the contrary, diphenyleneiodonium activated both PI3K/Akt and Erk signaling pathways without affecting Wnt. Our data indicates limits and possible unexpected interactions of NOXs inhibitors with intracellular signaling pathways.

## 1. Introduction

Reactive oxygen species (ROS) play multiple roles in the biology of the cell [1]. NADPH oxidase (NOX) and oxidative reactions on the mitochondrial membrane are the main sources of ROS, although it can also be produced by other enzymatic and nonenzymatic sources [2, 3]. NOX is a membrane-bound protein complex generating superoxide anion (O_2_^∙−^) from molecular oxygen which initiates the cascade of free radical reactions in response to various stimuli. Production of ROS by NOX family has long been considered a unique property of phagocytic cells, which utilize this enzyme as a part of host defense immune system. Currently, the regulated ROS production in nonphagocytic cells by NOX was linked to regulation of different processes including proliferation, migration, differentiation, immunomodulation, and oxygen sensing, and therefore, its expression and activity are tissue specific and are tightly controlled [4, 5]. On the basis of the homology to the catalytic subunit of the original phagocytic NOX (gp91phox or preferably NOX2), other NOX isoforms—NOX1, NOX3, NOX4 and NOX5—have been identified in nonphagocytic cells. In parallel, two other members of the NOX family were discovered, namely, dual oxidases 1 and 2 (DUOX 1, 2), initially also referred to as thyroid oxidases [6].

Despite studies suggesting the importance of NOXs in general, the involvement of individual NOX family members in specific function is still not completely understood. One of the reasons is a lack of highly specific inhibitors that would reliably block particular NOX. The most frequently used inhibitors employed in experiments are vanillin derivative 4-hydroxy-3-methoxyacetophenone (trivial names: apocynin or acetovanillone, APO) and diphenyleneiodonium chloride (DPI). Both of these drugs were applied in numerous* in vitro* and* in vivo* studies and although their effect was attributed primarily to NOX inhibition, specificity of APO and DPI remains questioned [7].

The proposed molecular mechanism of APO-mediated NOX inhibition is not fully understood but it involves impairment of NOX complex assembly and activation [8]. APO was also shown to act directly as ROS scavenger [9]. Contrarily to this finding, other studies suggest that APO is rather a prooxidant stimulating ROS production [10–12]. Further, APO was also shown to modulate the generation of arachidonic acid-derived inflammatory mediators [13].

DPI was reported not only to affect NOX, but also to interfere with other flavoenzymes, including nitric oxide synthase and xanthine oxidase [14, 15]. Inhibitory effects of DPI on mitochondrial ROS production were also shown [16]. On the other hand, DPI induces O_2_^∙−^ mediated apoptosis [17], inhibits cell redox metabolism, and promotes general oxidative stress [18]. DPI is also suggested as a nonselective blocker of ionic channels [19]. The above mentioned nonspecific effects are thought to be responsible for contradictory results obtained using these inhibitors. Further studies are needed to better understand the actions of APO and DPI not directly related to NOX inhibition.

Intracellular formation of ROS leading to overall redox status modulation is important for regulation of pluripotent cells differentiation [20]. In mouse embryonic stem cells (mES), pluripotent cells derived from inner cell mass of blastocyst, redox alterations are thought to play a role in the balance between self-renewal and differentiation. Undifferentiated mES have several times lower ROS level in comparison with differentiating mES [21]. It was shown that short term increase of the ROS favors differentiation into cardiomyocytes [22, 23] and into endoderm and mesoderm lineage [24].

Several signaling pathways are crucial for the regulation of self-renewal and differentiation of mES. Primarily, the mES pluripotency is controlled by Stat3 together with PI3K/Akt signaling pathways [25–27]. Importance of PI3K signaling was demonstrated in many studies where its inhibition negatively regulates the self-renewal in mES [28, 29]. Further, MAPK/Erk signaling pathway is rather important for mES differentiation as its inhibition improves maintenance of the pluripotent stem cell phenotype [30]. A growing body of evidence indicates that Wnt/*β*-catenin signaling pathway plays a vital role in the regulation of mES fate [31]. Signaling pathways including Stat3, PI3K/Akt, Erk, and Wnt are modified by ROS production [5, 32, 33] and therefore might be affected by NOX or other ROS modulating agents, although the importance of this phenomenon in mES remains elusive.

Our study demonstrates unexpected prooxidant activity of APO and DPI in undifferentiated mES together with impairment of cell proliferation. Further, we show that APO inhibits PI3K signaling accompanied by decrease in protein level of critical ES pluripotency regulator Nanog, whereas DPI enhances both PI3K and Erk activity. Interestingly, APO strengthens Wnt activity, pointing out another unknown mechanism of APO-mediated changes in signaling cascades regulating mES. In contrast to PI3K and Erk, we did not observe any effect of APO or DPI on Stat3 phosphorylation, which is considered to play the major role in mES maintenance.

## 2. Material and Methods

### 2.1. Cell Culture and Treatment

A feeder-free adapted mES line R1 was propagated as described previously [29] in an undifferentiated state by cell culturing on tissue culture plastic coated by gelatin (0.1% porcine gelatin solution in water) in Dulbecco's modified Eagle's medium (DMEM) containing 15% fetal bovine serum (FBS), 100 IU/mL penicillin, 0.1 mg/mL streptomycin, 1x nonessential amino acid (all from Gibco-Invitrogen, UK), and 0.05 mM *β*-mercaptoethanol (Sigma-Aldrich, USA), supplemented with 5 ng/mL of leukemia inhibitory factor (LIF, Chemicon, USA) referred to here as the complete medium.

APO, DPI, hydrogen peroxide (H_2_O_2_), N-acetylcysteine (NAC), and LY294002 (LY) were provided from Sigma-Aldrich. Stock solutions of APO (0.4 M), DPI (10 mM), and LY (10 mM) were prepared by dissolving the compounds in dimethyl sulfoxide. Aliquots were stored at −20°C. NAC was prepared as a 0.5 M stock solution in serum-free DMEM medium, pH was adjusted to 7.4, and filter-sterilized aliquots were stored at −20°C. Drugs were added directly to the incubation medium or freshly prediluted in sterile phosphate-buffered saline (PBS) to desired concentration.

### 2.2. Cell Proliferation

The cell proliferation was determined by estimation of overall cellular protein mass in whole cell lysates that reflects the cell number as demonstrated previously [34]. ES cells were seeded to 24-well plate in complete media at density 5 000 cells per cm^2^. Next day, the cells were treated with drugs for further 48 hours. Finally, the cells were washed twice with PBS and lysed in SDS buffer (50 mM Tris-HCl, pH 7.5, 100 mM NaCl, 10% glycerol, 1% SDS, 1 mM EDTA). Protein concentration was determined using DC protein assay (Bio-Rad, USA) kit according to manufacturer's instructions.

### 2.3. Determination of NOX Expression

Total RNA was extracted using the UltraClean Tissue & Cells RNA Isolation Kit (MO BIO Laboratories, USA) for ESC and RNAzol RT (Molecular research center Inc., USA) for mouse tissues according to manufacturer's instructions. 1 *μ*g of total RNA was used for cDNA synthesis with DyNAmo cDNA Synthesis Kit (Finnzymes, Finland) according to the manufacturer's instructions. qPCR was performed in LightCycler 480 instrument using LightCycler Probes Master with Universal Probe library probes (all from Roche, Germany) according to manufacturer's instructions. Ribosomal protein L13A (RPL13A) was used as reference gene; data are presented as 2^−(Cq(target)−Cq(reference))^. The primers and probes used were as follows:

NOX1: 5′tggattttctaaactaccgtctcttc, 5′caaagtttaatgctgcatgacc3′, #20; NOX2: 5′tgccaacttcctcagctaca3′, 5′gtgcacagcaaagtgattgg3′, #20; NOX3: 5′tgaacaagggaaggctcatt3′, 5′catcccagtgtaaagctatgtga3′, #20; NOX4: 5′catttgaggagtcactgaactatga3′, 5′tgtatggtttccagtcatccag3′, #5; DUOX1: 5′acagatggggcagcaaag3′, 5′gctggtagacaccatctgcat3′, #20; DUOX2: 5′cagacagctttttgctcaggt3′, 5′cacttgctgggatgagtcc3′, #64; RPL13A: 5′catgaggtcgggtggaagta3′, 5′gcctgtttccgtaacctcaa3′, #25.

### 2.4. Analysis of ROS Production in Live Cells by Automated Time-Lapse Image (Live Imaging Fluorescent Microscopy)

The cells were seeded to 96-well plate designed for live imaging fluorescence microscopy (Greiner Bio-one, Germany). After 24 hours, cells were pretreated with tested drugs for 15 minutes and loaded with hydroethidine (HE, 5 *μ*M, Sigma-Aldrich). Live imaging of prepared samples was performed at 37°C and 5% CO_2_ atmosphere using the high-content screening microscope ImageXpressMicroXL (Molecular Devices, USA). Seven images per well were acquired during 120 minutes with scanning interval of 10 minutes. By Gaussian thresholding of the HE fluorescence images, mask area of the viewfield covered with metabolizing cells was detected using the Otsu method of fitting individual objects. The 10640 images for every measured plate were analyzed with CellProfiler [35], running on processor cluster provided by MetaCentrum (The National Grid Infrastructure). Intensity of fitted regions was lowered by minimal intensity of an appropriate image to subtract the background and the mean intensity per individual well was then calculated and visualized with Knime [36], using HCS tools and R Statistics Integration extensions [37].

### 2.5. High-Performance Liquid Chromatography (HPLC) Analysis of ROS Production

The HPLC detection of O_2_^∙−^ was based on the detection of a specific product 2-hydroxyethidium 2-OH-E(+) which is formed in the reaction of O_2_^∙−^ with HE [38, 39]. Besides specific 2-OH-E(+), also a nonspecific product of hydride acceptors with HE – ethidium (E+) was detected. The cells were seeded to 6-well plate and before the treatment the medium was changed for DMEM (without phenol red and sodium pyruvate) with 1% FBS. The cells were treated with APO and DPI for 120 minutes in total; 30 minutes before the end of the experiment HE in final concentration 10 *μ*M was added. The medium after centrifugation was stored for optional HPLC analysis. To extract the HE products, ice cold methanol was added to the cells [40] for 15 minutes at 4°C in dark, shaking. The supernatant was transferred to an Eppendorf tube and centrifuged. A 75 *μ*L sample was injected into the HPLC system (Agilent series 1100) equipped with fluorescence and UV detectors (Agilent series 1260) to separate the 2-OH-E(+) product. Fluorescence was detected at 510 nm (excitation) and 595 nm (emission). The mobile phase consisted of H_2_O/CH_3_CN. Kromasil C18 (4.6 mm × 250 mm) column was used as the stationary phase. Elution conditions for the analysis of HE and its products were used from Nature Protocols [38].

### 2.6. Western Blot Analysis

Western blot analysis, cell sample harvesting, and preparation were performed by a standard procedure as presented previously [29]. We used the following primary antibodies against Nanog, *β*-actin (Abcam, USA), p-Akt (S473), Akt, p-Stat3 (Y705), Stat3, Erk1/2, p-Erk1/2 (T202/Y204), and p-GSK3*β* (S9) (all from Cell Signaling Technology, USA). Following immunodetection, each membrane was stained by amido black to confirm the transfer of the protein samples. The total level of *β*-actin was detected as loading control.

### 2.7. Cell Transfection and TOPflash Luciferase Reporter Assay

Cells were transiently transfected using polyethyleneimine in a stoichiometry of 4 *μ*L per 1 *μ*g of DNA. Super8X TOPflash construct,* Renilla* luciferase construct, and expression vector for mutant nondegradable *β*-catenin, and S33-*β*-catenin (codon 33 substitution of Y for S, generously provided by Professor Korswagen) were used in concentration of 0.5 *μ*g per well in a 24-well plate 24 hours after seeding. 6 hours after transfection medium was changed and cells were treated with NOX inhibitors or LY for 24 hours. For cell stimulation Wnt3a or control conditioned medium [41] was added 8 hours before harvest. Dual-Luciferase assay kit (Promega, USA) was used according to the manufacturer's instructions for the evaluation of luciferase activity. Relative luciferase units were measured on a plate luminometer Chameleon V (Hidex, Finland) and normalized to the* Renilla* luciferase expression.

### 2.8. Statistical Analysis

Data are expressed as mean + standard error in the mean (SEM). Statistical analysis was assessed by *t*-test or by one-way analysis of variance ANOVA and Bonferroni's Multiple Comparison posttest. The values of *P* < 0.05 were considered statistically significant (^*∗*^ *P* < 0.05, ^*∗∗*^ *P* < 0.01, and ^*∗∗∗*^ *P* < 0.001).

## 3. Results

### 3.1. Expression of NOXs in mES Is Relatively Low

**Figure 1:**
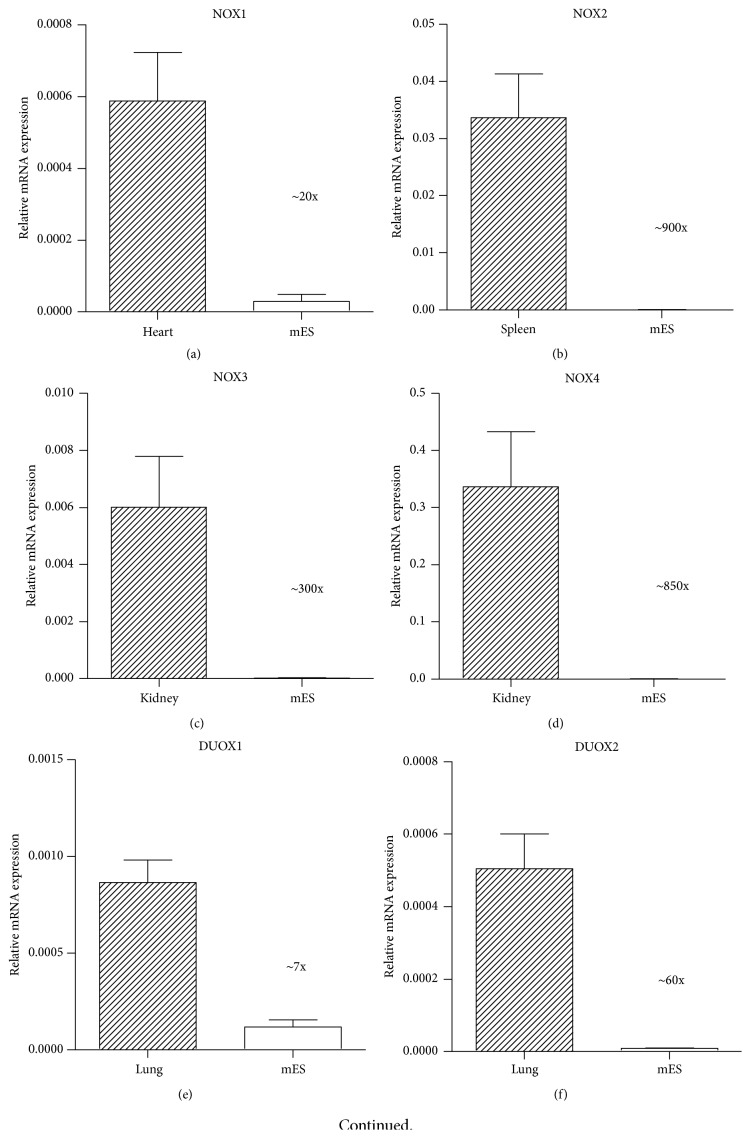
Relative expression of NOX1 (a), NOX2 (b), NOX3 (c), NOX4 (d), DUOX1 (e), and DUOX2 (f) mRNA in mES and selected tissues. Comparison of relative expression of individual NOXs and DUOXs mRNA within mES cells is also shown (g). Data are presented as mean + SEM from at least two independent experiments.

To confirm the assumption of the negligible presence of NOX and DUOX homologues in the undifferentiated mES, the gene expression was compared to the selected mouse tissues. Particular organs were chosen based on the described presence of NOX homologues by various authors [4, 6]. In agreement with the literature, all determined NOX homologues NOX1, NOX2, NOX3, NOX4, DUOX1, and DUOX2 showed two to three orders lower expression in undifferentiated mES compared to selected tissues (Figures 1(a)–1(f)). The comparison of the NOXs relative expression in mES revealed the NOX4 expression to be the highest, approximately 10 times compared to other NOXs (Figure 1(g)), altogether, it can be concluded that the expression of all NOXs except NOX4 is very low.

### 3.2. APO and DPI Affect mES Proliferation

**Figure 2:**
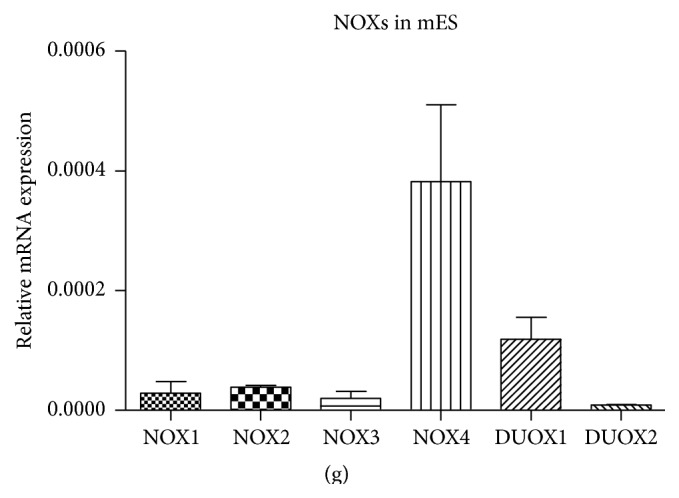
Effect of APO (a) and DPI (b) on mES proliferation after 48 h treatment based on total cellular protein mass. Data represent mean + SEM from four independent experiments. Statistical significance was determined by ANOVA post hoc Bonferroni's Multiple Comparison test, *P* < 0.05. The groups marked differently by symbol letters are statistically significantly different from each other.

Both APO and DPI affected growth of mES in dose-dependent manner in the concentration range 0.1, 0.25, 0.5, 1.0, and 2.0 mM for APO (Figure 2(a)) and 10, 20, 40, 80, 160, and 320 nM for DPI (Figure 2(b)). Data showed decrease in cell proliferation using concentration from 0.5 mM (APO) and from 20 nM (DPI) after 48-hour treatment. IC50 of APO and DPI for mES determined from these data were 1.1 ± 0.2 mM and 18.5 ± 3.2 nM, respectively.

### 3.3. APO and DPI Do Not Inhibit but Potentiate Formation of ROS in mES

**Figure 3:**
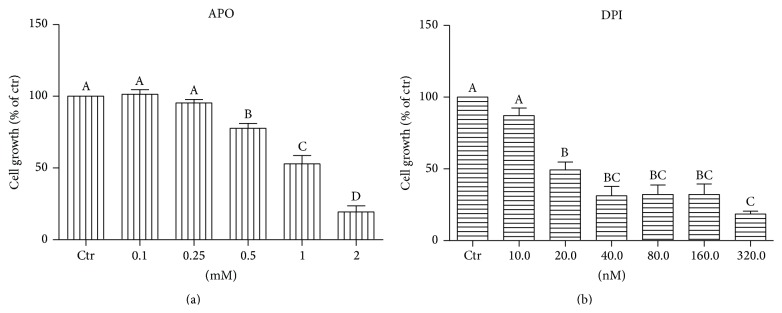
ROS production in mES cells treated by 1 mM APO and 100 nM DPI computed from the automated time-lapse image acquisition of HE fluorescence for 120 minutes (a, b); selected single time point 60 minutes (c). HPLC determination of specific 2-OH-E(+) and nonspecific product E(+) of HE oxidation in the presence of 1 mM APO and 100 nM DPI (d, e). Data are presented as mean + SEM from four independent experiments. Statistical significance was determined by ANOVA post hoc Bonferroni's Multiple Comparison test, *P* < 0.05. The groups marked by an asterisk are statistically significantly different from control.

Despite the very low expression of NOXs enzymes in undifferentiated mES, the ROS production was detectable in our mES lines. Contrary to the expectations, treatment by APO or DPI significantly induced generation of ROS in mES cells detected by live imaging analysis of HE fluorescence, continuously for up to 120 minutes (Figures 3(a), 3(b), and 3(c)). To confirm the specificity of this determination, the formation of specific product of HE reaction with O_2_^∙−^, the 2-OH-E(+), and also nonspecific product, ethidium (E+), were determined by HPLC (Figures 3(d) and 3(e)). This analysis shows potentiation of nonspecific HE oxidation rather than O_2_^∙−^ formation in DPI treated cells, but not in APO treated cells.

### 3.4. Effect of APO and DPI on Stat3, Akt, and Erk Phosphorylation in mES

**Figure 4:**
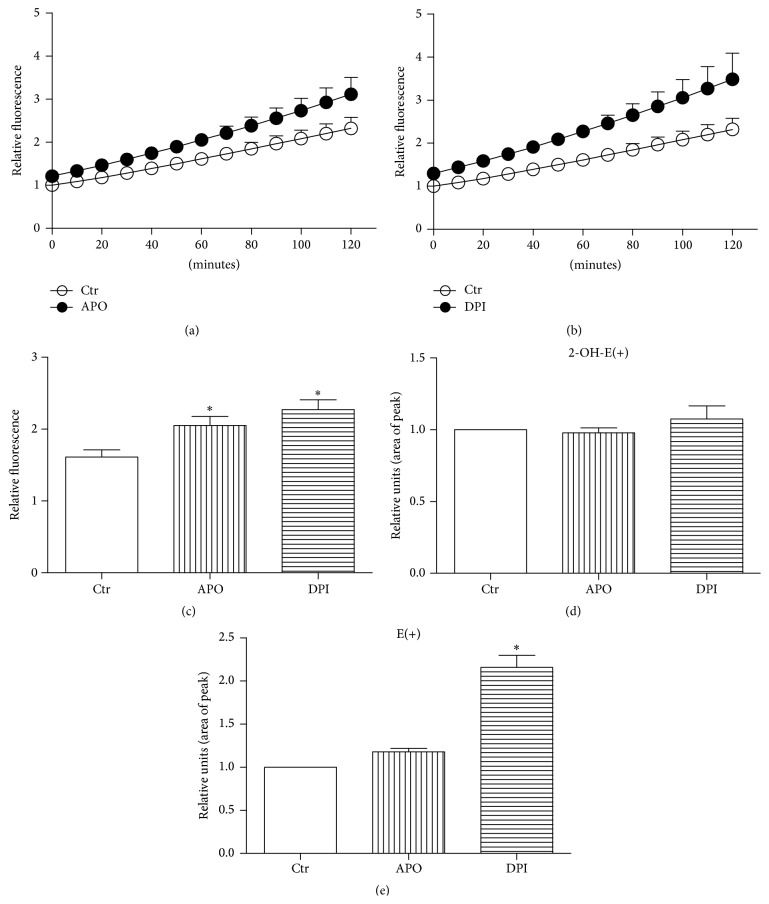
Effect of APO (2 mM) and DPI (100 nM) on the Stat3, Akt, and Erk phosphorylation in serum starved mES cells (SF) treated by drugs for 20 minutes followed by LIF (5 ng/mL) or FBS (15%) stimulation for 20 minutes. Total level of *β*-actin was used as a loading control. A typical representative western blot is shown.

To investigate the effect of short term treatment, mES were serum and LIF starved for 12 hours and treated by APO and DPI for 20 minutes followed by 20-minute stimulation with FBS or LIF. APO treatment resulted in decrease of Akt phosphorylation in every condition without effect on Erk. DPI had no significant effect on Akt and Erk kinases signaling. Level of p-Stat3 remained unchanged by NOX inhibitors (Figure 4).

To further clarify the dose-dependent effect of tested drugs, we employed the same experimental design with serum starvation and FBS activation. Moreover, glutathione precursor NAC and H_2_O_2_ were used as a* bona fide* antioxidant and a prooxidant, respectively, to distinguish between effects mediated via redox changes in cultivated mES and ROS independent actions of APO and DPI.

**Figure 5:**
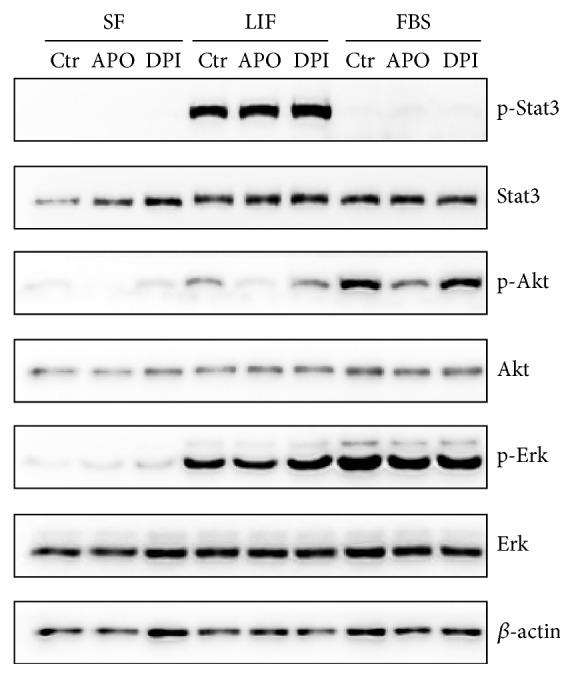
Effect of APO (1, 2, 4 mM), DPI (0.1, 1, 100 *μ*M), NAC (5, 10, 20 mM), and H_2_O_2_ (0.5, 1 mM) on the Akt and Erk phosphorylation in serum starved mES cells treated by drugs for 20 minutes followed by 15% FBS stimulation for 20 minutes (a) and cells treated for 1 hour in complete medium (b). Total level of *β*-actin was used as a loading control. A typical representative western blot is shown.

APO abolished phosphorylation of Akt in a dose-dependent manner with supportive effect on phosphorylation of Erk in higher concentrations. In contrast, DPI slightly increased phosphorylation of Akt and also upregulated phosphorylation of Erk in the highest concentration. Consistent with expectations, H_2_O_2_ increased both Akt and Erk phosphorylation. NAC had no effect on signaling in this setup (Figure 5(a)). To test whether the effect on signaling was also preserved during cultivation in complete medium, mES were treated by the same concentration of drugs for 1 hour. In this case, similarly to previous treatment, APO inhibited Akt phosphorylation in a dose-dependent manner. Erk phosphorylation was impaired in the presence of the highest concentration of APO. Notably, in this setup 100 *μ*M concentration of DPI strongly induced both Akt and Erk phosphorylation. Contrary to the previous setup, NAC decreased phosphorylation of both pathways (Figure 5(b)).

**Figure 6:**
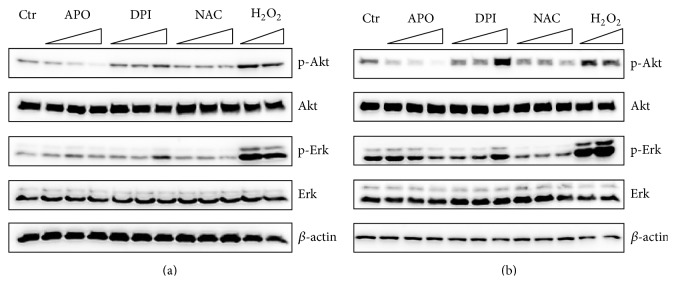
Effect of APO (1 mM) and DPI (10 nM) in absence and presence of antioxidant NAC (10 mM) on the Akt and Erk, phosphorylation and Nanog protein level in mES cells after 24 hours in complete medium. Total level of *β*-actin was used as a loading control. A typical representative western blot is shown.

Finally, we examined the impact of APO and DPI on Akt and Erk activity after 24 hours in absence or presence of NAC (Figure 6). Contrary to the effect observed after a short term treatment, Akt phosphorylation was upregulated when APO was employed. Phosphorylated form of Akt was also increased following the DPI treatment. This activation could be prevented by addition of NAC. Erk phosphorylation was slightly decreased by APO treatment and augmented by DPI even in the presence of NAC. APO strongly downregulated Nanog protein level in mES, independently of NAC supplementation (Figure 6). Level of Stat3 phosphorylation was modulated in the above mentioned experimental condition by neither APO nor DPI treatment (data not shown). We did not observe any effect of H_2_O_2_ on the evaluated signaling pathways after 24 hours (data not shown).

### 3.5. APO Augments Canonical Wnt Activity in mES

**Figure 7:**
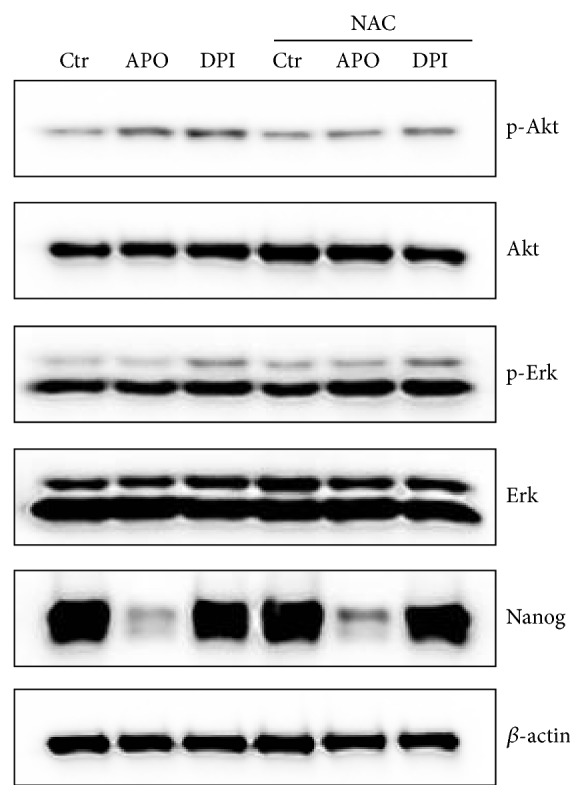
Effect of APO and DPI on Wnt pathway activity determined by TOPflash assay and GSK3 phosphorylation on S9. Effect of the Wnt3a conditioned media or exogenous nondegradable *β*-catenin on the transcriptional activation of a reporter gene (a). Effect of LY (10 *μ*M), APO (1 mM), and DPI (10 nM) on the spontaneous (b), Wnt3a conditioned media induced (c), and exogenous nondegradable *β*-catenin-induced transcriptional activation (d). Data represent mean + SEM, from at least four independent experiments. Statistical significance was determined by ANOVA post hoc Bonferroni's Multiple Comparison test (^*∗*^ *P* < 0.05, ^*∗∗*^ *P* < 0.01, and ^*∗∗∗*^ *P* < 0.001). Effect of LY, APO, and DPI on GSK3*β* (S9) phosphorylation (e). Total level of *β*-actin was used as a loading control. A typical representative western blot is shown.

To assess the effects of APO and DPI supplementation on activity of Wnt pathway, we used TCF/LEF reporter gene assay (TOPflash) to determine the level of canonical Wnt activation mediated by *β*-catenin which specifically induces the transcriptional activity of TCF/LEF [42]. Firstly, we examined our system with addition of Wnt3a conditioned media or exogenous nondegradable *β*-catenin, both known agonists, to promote its activation. These interventions induced transcription activity of reporter gene 45 and 25 times, respectively (Figure 7(a)).

PI3K inhibitor LY294002 (LY) and both NOXs inhibitors APO and DPI did not change the spontaneous *β*-catenin mediated transcription activity of TCF/LEF (Figure 7(b)). In contrast, when the cells were treated by Wnt3a conditioned media, presence of LY and APO significantly augmented the TCF/LEF transcription activity (Figure 7(c)). On the other hand, DPI had no effect (Figure 7(c)). However, all tested drugs did not have any effect in the presence of exogenous nondegradable *β*-catenin (Figure 7(d)).

To further clarify the effects of LY, APO, and DPI on Wnt/*β*-catenin signaling, the GSK3*β* S9 phosphorylation, allowing accumulation of *β*-catenin, was evaluated [43]. Treatment with both APO and LY but not DPI decreased GSK3*β* S9 phosphorylation in this setup as shown by the western blot analysis (Figure 7(e)).

## 4. Discussion

APO and DPI are the most commonly used inhibitors of NOX, involved in numerous studies despite the increasing evidence questioning their specificity. We employed mES as a model to analyze effects of these compounds that might not be directly mediated through impairment of NOXs, because of their generally negligible expression in undifferentiated mES. We aimed to investigate modulation of ROS production by APO and DPI in mES as well as interactions with signaling pathways important for stem cell regulation.

Although NOXs were attracting attention as an important source of intracellular ROS production for a long time, their role in stem biology remains poorly understood. Previously, it was demonstrated that NOXs expression is precisely regulated during embryonic stem cell differentiation into cardiomyocytes [44, 45] and vascular smooth muscle lineage [46].

In our experiments, we observed generally low level of NOXs/DUOXs expression close to the limit of detection, which we concluded both from real Cq values of PCR amplification and from comparison of NOXs/DUOXs expression in several employed tissues. As such control sample, tissues with well described NOXs/DUOXs expression and activity were used [4, 6]. Among NOXs and DUOXs, NOX4 expression was the highest in mES, which is in agreement with other authors [46, 47]. It may also correspond to relative abundant expression of this enzyme across other tissues [48]. However, it should be emphasized that level of NOX4 transcript in mES was still nearly three orders below expression in selected control tissue (kidney). NOX4 was shown to be constitutively active, and hence its activity is mostly regulated by the level of its expression [49]. Concerning other potential sources of intracellular ROS, it is also noteworthy that favored reliance on anaerobic glycolysis in undifferentiated mES leads to reduced mitochondrial biogenesis and activity, manifested by declined ROS production [50, 51]. Thus mES can be considered not only NOX-low, but also overall ROS-low model.

Next, we aimed to assess effect of APO and DPI on proliferation of undifferentiated mES. Selection of used concentrations was based on comprehensive literature search. APO as NOX inhibitor was used in range 30–1200 *μ*M [9, 52] with the upper range of these concentrations corresponding to APO supplementation preferably employed in our experiments. Regarding DPI, many authors used concentrations ranging 1–100 *μ*M {summarized in [53]} which is approximately one order of magnitude higher than doses used in our experiments, as we observed significant growth impairment even when concentrations as low as 20 nM were applied. Impact on cell proliferation after NOX inhibitors treatment was described earlier, for both normal and transformed cell lines. The observed effects of DPI and APO were suggested to be attributed to the various mechanisms including changes in NOX mediated ROS production, downregulation of integrin expression, cell cycle arrest, or modulation of mitogenic-signaling pathways [54–57]. Therefore, we can assume that direct modulation of ROS production could contribute to the observed decrease of mES proliferation. At the same time, the effects of inhibitors employed in this study can also be related to their direct effects on the other promitogen cell signaling pathways as discussed later. The potential of APO and DPI to inhibit cell proliferation could also be beneficial in the context of anticancer agents. A recent publication showed that APO suppressed prostate cancer and that the reduction of Rac1 and NF*κ*B phosphorylation was involved [58].

Further, we assessed how NOX inhibitors affected ROS production in our model. Due to their natural short half-life and high reactivity, precise detection of ROS represents a tremendous challenge, especially in nonphagocytic cells. We were aware of limits in ROS measurement with respect to specificity and generation of possible artefacts; therefore, we used two different assays, live imaging fluorescent microscopy and HPLC, both utilizing ROS-sensitive probe HE. The reaction between O_2_^∙−^ and HE generates a highly specific red fluorescent product 2-hydroxyethidium 2-OH-E(+). However, in the intracellular milieu, the presence of redox metal ions or hemeproteins with peroxidase activity or other one-electron oxidants can oxidize HE to several nonspecific products, including the ethidium E(+) and dimeric products [38, 59, 60]. Despite the expectations, the live imaging assay showed significant prooxidant activity of APO and DPI when continuous nonoscillating probe oxidation was determined by live imaging. On the other hand, HPLC analysis used for determination of specific HE derivatives did not confirm O_2_^∙−^ production after APO and DPI treatment. The only observed effect was significant DPI-mediated elevation of E(+). APO did not induce generation of HE oxidation products {both 2-OH-E(+) and E(+)}, which is partially in contrast with the results from live imaging fluorescent microscopy where all nonspecific HE oxidation fluorescent products are summarized [60]. This suggests that other oxidants, rather than O_2_^∙−^, are responsible for increase in HE fluorescence.

Although NOX inhibitors should generally relieve oxidative stress, several lines of evidence for APO and DPI exist demonstrating the opposite. It was reported that DPI inhibits pentose phosphate pathway (PPP) responsible for the synthesis of NADPH, a redox cofactor important for many antioxidant enzymes, thus making the cells more prone to oxidative stress [18]. Suggested mechanism included direct inhibition of NADP-dependent enzymes such as glucose 6-phosphate dehydrogenase, glyceraldehyde 3-phosphate dehydrogenase, and lactate dehydrogenase. In agreement with our data, evidence from different group exists, showing that DPI is exerting prooxidative effects in given cell types and conditions [17]. Further, it was reported that APO, contrary to DPI, stimulates PPP which is known to be a subsequent step following oxidative stress exposure. This was prevented by addition of GSH into medium, further suggesting that APO-induced GSH oxidation might be involved in observed PPP activation [11]. However, in a different study APO opposingly increased the synthesis of the GSH through activation of transcription factor AP-1. Notably, levels of GSSG were not altered, implying that APO itself does not cause oxidative stress [61]. Many studies are highlighting that APO is a prodrug that must be first metabolized through oxidation to its oligomeric form; thus certain redox environment must be present in order to achieve full APO activation [62] narrowing its function preferably to cells with strong ROS production like stimulated endothelial cells [63] or professional phagocytes [64]. In compliance with these findings, it was demonstrated that APO can act both as an antioxidant and as a prooxidant, depending on the cell type and its oxidizing potential [9, 10]. This is in agreement with our experiments performed on nonphagocytic low-level ROS cells where APO might rather contribute to oxidative stress as demonstrated by live imaging. Interestingly, different modifications of APO are suggested for* in vivo* experiments [65], further revealing the complexity of this issue.

Critical features of mES, pluripotency, self-renewal, and unlimited proliferation, are predominantly exerted through actions of cytokine LIF, which is routinely added to the culture medium. Binding of LIF to its receptor triggers the activity of three major intracellular signaling cascades: JAK/Stat3, PI3K/Akt, and MAPK/Erk. These pathways converge to regulate the gene expression pattern typical for mES [27]. To clarify possible effects of APO and DPI on selected signaling pathways, the modulation of Stat3, Akt, and Erk activation status was analyzed in mES cells. Firstly, mES were serum and LIF depleted in order to increase cellular response, as cultivation in complete medium leads to a constant activation of those pathways. Stimulation by LIF and FBS was employed to reactivate signaling [25, 66]. As expected, LIF addition induced preferably Stat3 response while FBS, containing growth factors and cytokines, augmented Akt and Erk signaling. To further analyze effect of drugs on selected kinases, we examined their phosphorylation status also in complete medium after 1-hour and 24-hour treatment, to elucidate early changes and later cell response in signaling pathways. In agreement with other studies, both Akt and Erk kinases responded in ROS-sensitive manner [67, 68], which we demonstrated by H_2_O_2_-induced phosphorylation. Similarly, we can assume that DPI-mediated ROS production is responsible for the observed effects on Akt and Erk activation which is in contrast to the response to APO. Properties of general antioxidant NAC in sense of attenuation of kinase phosphorylation were more profound during treatment in complete medium. Notably, we did not observe changes of Stat3 phosphorylation in presence of any drugs tested in our study. The most striking effect was detected downstream of PI3K on the level of S473 Akt phosphorylation when APO treatment was employed. In serum starved cells, in FBS or LIF activated cells, and also in the presence of complete medium, APO decreased Akt phosphorylation when short term impact was studied. It was earlier reported that vanillin and some of its derivatives, including APO, readily inhibited PI3K in lung adenocarcinoma cell line [69]. These authors are also suggesting, in agreement with our data, that radical scavenging or other antioxidant properties of those compounds are not responsible for the observed effect. Thus, the mode and mechanism of inhibition needs to be further clarified.

Remarkably, critical role of PI3K was also described for the process of NOX activation [70]; thus it can be hypothesized that some of the APO inhibitory actions towards NOXs might be also mediated* via* PI3K inhibition. To further elucidate impact of APO-induced PI3K inhibition, we examined the level of homeodomain transcription factor Nanog, which was suggested as a downstream target of PI3K and is also recognized as an important intrinsic regulator of stem cell pluripotency [28]. After 24 hours of APO treatment, the level of Nanog was dramatically reduced. Interestingly Akt phosphorylation was upregulated by APO after 24 hours compared to untreated cells, and this increase could be reverted by addition of NAC, although Nanog levels remained diminished, suggesting that this impairment is perhaps not related to APO-induced modulation of ROS levels in the cell. Phosphorylation of both Akt and Erk was augmented by DPI treatment that may be attributed to its above described prooxidative effect, as it was reduced by NAC supplementation in the case of Akt.

Next, we examined the impact of NOX inhibitors on canonical Wnt signaling, as it also plays a distinctive role in regulation of embryonic stem cells [31, 71] and the modulator of this pathway, GSK3, is a known PI3K downstream [72]. GSK3 regulates Wnt pathway by phosphorylating *β*-catenin on multiple sites that enhances its subsequent degradation [43]. GSK3 is active in resting cells but is readily inhibited through the PI3K/Akt-mediated phosphorylation of N-terminal serine residues (S9 in GSK3*β* and S21 in GSK3*α*) [72]. Therefore, in the conventional view, it is assumed that activity of PI3K/Akt should promote canonical Wnt signaling. In contrast to this, experimental evidence argues against this simplified scenario, as Akt activation failed to promote Wnt/*β*-catenin signaling when insulin and constitutively active Akt were administrated [73]. Moreover, it was reported that Axin-associated GSK3, responsible for mediating *β*-catenin degradation, represents just a minor fraction of GSK3 cellular content and this complex is not accessible to Akt phosphorylation, thus preventing PI3K/Akt/Wnt cross-talk [74]. In our experiments, we did not see any effect on basal Wnt transcription activity, when NOX inhibitors or LY was employed. Moreover, treatment of both APO and LY decreased GSK3*β* S9 phosphorylation, further questioning the simplified model of PI3K/Wnt cross-talk mediated solely by level of GSK3 inhibition. However, after Wnt3a induction, both LY and APO augmented Wnt transcription activity. Notably, PI3K inhibition was recently shown to increase the amount of active nuclear *β*-catenin and to promote induction in TOPflash assay in epithelial cell culture system [75]. In agreement with other studies [76, 77], authors were suggesting the pivotal role of bidirectional loop between receptor tyrosine kinase-driven MAPKs and Wnt/*β*-catenin signaling. Although Erk kinase was not dramatically affected by APO treatment in our system, we are not excluding the possibility of a different MAPK involvement in observed phenomena, as numerous convergence points were described between PI3K and MAPKs [78].

## 5. Conclusions

Altogether, our study suggests prooxidant activity of APO and DPI in mES. Moreover, treatment with those drugs results in different modulation of intracellular pathways critical for regulation of proliferation and differentiation. APO markedly downregulates activity of Akt and its downstream Nanog and augments Wnt signaling. DPI promotes Akt and Erk activation. Taking into account described negligible NOXs levels in mES we suggest that actions of drugs observed in our experiments are rather independent of their typical function as NOX inhibitors. Therefore, caution should be taken to potential applications of these NOX inhibitors and interpretation of obtained results, especially in studies focused on the stem cell biology and intracellular and redox signaling.
